# Cutaneous lesions and other non-endocrine manifestations of Multiple Endocrine Neoplasia type 1 syndrome

**DOI:** 10.3389/fendo.2023.1191040

**Published:** 2023-07-07

**Authors:** Laura Pierotti, Elena Pardi, Elisa Dinoi, Paolo Piaggi, Simona Borsari, Simone Della Valentina, Chiara Sardella, Angela Michelucci, Maria Adelaide Caligo, Fausto Bogazzi, Claudio Marcocci, Filomena Cetani

**Affiliations:** ^1^ Department of Clinical and Experimental Medicine, University of Pisa, Pisa, Italy; ^2^ Department of Information Engineering, University of Pisa, Pisa, Italy; ^3^ Unit of Endocrinology, University Hospital of Pisa, Pisa, Italy; ^4^ Laboratory of Molecular Genetics, University Hospital of Pisa, Pisa, Italy

**Keywords:** angiofibroma, lipoma, primary hyperparathyroidism, pituitary, adrenal, pancreas, GEP, cutaneous lesions

## Abstract

**Background:**

Multiple Endocrine Neoplasia type 1 is a rare genetic syndrome mainly caused by mutations of *MEN1* gene and characterized by a combination of several endocrine and non-endocrine manifestations. The objective of this study was to describe cutaneous lesions and other non-endocrine manifestations of *MEN1* in a cohort of patients with familial (F) and sporadic (S) *MEN1*, compare the prevalence of these manifestations between the two cohorts, and investigate the correlation with *MEN1* mutation status.

**Methods:**

We collected phenotypic and genotypic data of 185 patients with F-*MEN1* and S-*MEN1* followed from 1997 to 2022. The associations between F-*MEN1* and S-*MEN1* or *MEN1* mutation-positive and mutation-negative patients and non-endocrine manifestations were determined using chi-square or Fisher’s exact tests or multivariate exact logistic regression analyses.

**Results:**

The prevalence of angiofibromas was significantly higher in F-*MEN1* than in S-*MEN1* in both the whole (p < 0.001) and index case (p = 0.003) cohorts. The prevalence of lipomas was also significantly higher in F-*MEN1* than in S-*MEN1* (p = 0.009) and in *MEN1* mutation-positive than in *MEN1* mutation-negative (p = 0.01) index cases. In the whole cohort, the prevalence of lipomas was significantly higher in *MEN1* mutation-positive compared to *MEN1* mutation-negative patients (OR = 2.7, p = 0.02) and in F-*MEN1* than in S-*MEN1* (p = 0.03), only after adjustment for age. No significant differences were observed for the other non-endocrine manifestations between the two cohorts. Hibernoma and collagenoma were each present in one patient (0.5%) and meningioma and neuroblastoma in 2.7% and 0.5%, respectively. Gastric leiomyoma was present in 1.1% of the patients and uterine leiomyoma in 14% of women. Thyroid cancer, breast cancer, lung cancer, basal cell carcinoma, melanoma, and colorectal cancer were present in 4.9%, 2.7%, 1.6%, 1.6%, 2.2%, and 0.5% of the whole series, respectively.

**Conclusions:**

We found a significantly higher prevalence of angiofibromas and lipomas in F-*MEN1* compared with S-*MEN1* and in *MEN1* mutation-positive compared to *MEN1* mutation-negative patients. In patients with one major endocrine manifestation of *MEN1* , the presence of cutaneous lesions might suggest the diagnosis of *MEN1* and a possible indication for genetic screening.

## Introduction

Multiple endocrine neoplasia type 1 (MEN1) is a rare hereditary syndrome with an estimated prevalence of approximately one to three in 100,000 inhabitants (1). In most patients (90%), MEN1 occurs in a familial form with an autosomal dominant inheritance. Mutations of *MEN1* gene are identified in up to 90% of index cases with familial disease and in up to 30% of sporadic cases (2). *MEN1* gene, consisting of 10 exons, is located on the long arm of chromosome 11 (11q13). The gene product, menin, plays a key role in the regulation of cell proliferation and differentiation by interacting directly or indirectly with more than 50 different proteins involved in cell adhesion, cell cycle progression, cell division, DNA repair, and other several signaling pathways (3–6). More than 1,500 mutations have been identified in familial and sporadic cases, without a correlation between genotype and phenotype (7, 8). Heterozygous germline inactivating mutation in *MEN1* gene represents the first hit usually followed by a second hit represented by the loss of a large chromosomal region (11q13) of the normal copy of the gene (LOH) in MEN1-associated endocrine tissues or another somatic mutation. Such events lead to the complete loss of function of the encoded protein menin, according to Knudson’s model of tumor suppressor genes (9).

A combination of more than 20 endocrine and non-endocrine manifestations has been reported in the affected subjects. Clinical variability has also been observed in subjects within the same family, suggesting that epigenetic regulation may contribute to the clinical phenotype of MEN1 (10). MEN1 syndrome most frequently involves the parathyroids, pancreatic islets, and pituitary (11). The most common manifestation is primary hyperparathyroidism (PHPT), which occurs in 99% of patients by the age of 50, usually caused by benign, uniglandular or multiglandular, synchronous or asynchronous, parathyroid involvement and extremely rarely by parathyroid carcinoma (5, 12). PHPT is the first manifestation of MEN1 in approximately 80%–85% of patients. Tumors of the gastroenteropancreatic (GEP) tract, present in 30%–70% of patients, typically occur after 40 years of age as non-functioning or functioning lesions secreting gastrin, insulin, glucagon, or vasoactive intestinal peptide (5). Pituitary involvement is present in 30%–40% of cases, mostly due to prolactinomas (5).

*MEN1* mutations seem to be a discriminating factor associated with the classic phenotype of MEN1 syndrome, whereas most of the *MEN1* mutation-negative patients have a different phenotype and clinical course of the disease, representing the so-called phenocopies (13). These patients, mainly affected by MEN1 without a familial history, have a later onset of the first manifestation, a lower likelihood of developing a third MEN1-related lesion, and a life expectancy comparable with that of the general population (2, 8, 13–15). In these cases, mutations of other genes might be responsible for a MEN1-like phenotype. In particular, mutations of *CDKN1B* gene encoding the cyclin-dependent kinase inhibitor p27 are responsible for the MEN4 syndrome (16, 17), whereas mutations of other cyclin-dependent kinase inhibitor (CDKI) genes—*CASR*, *AIP*, and *CDC73* genes—can also found in rare cases of MEN1-like phenotypes (2, 18, 19).

Several non-endocrine manifestations such as lipomas, angiofibromas, collagenomas, hibernomas, leiomyomas, and central nervous system tumors (meningiomas and ependymomas) have been reported in MEN1 patients (20–24). The association between cutaneous lesions and MEN1 syndrome was first reported in 1997 (20), and the prevalence of these manifestations differs in different series with a prevalence of 22%–88% of multiple facial angiofibromas, 0%–72% of collagenomas, and 5%–34% of lipomas (20–22, 25, 26). The finding of these lesions in association with endocrine tumors suggests the diagnosis of MEN1 syndrome. In particular, the occurrence of multiple angiofibromas as isolate cutaneous manifestation has the highest specificity, whereas a combination of multiple angiofibromas and any collagenomas has the highest sensitivity and specificity for MEN1 (22). Therefore, a thorough skin examination should be performed in patients with PHPT, GEP, and pituitary tumors, and the finding of cutaneous lesions raises suspicion of MEN1.

Increased risk and an early-onset of breast cancer have been reported in *MEN1*-mutated women than in the general population (27–29), and therefore, breast cancer surveillance should be started 10 years earlier in the former than in the latter women (27).

The objective of this study was to describe cutaneous lesions and other non-endocrine manifestations of MEN1 in a well-characterized cohort of patients with familial and sporadic MEN1 syndrome, compare their prevalence in the two cohorts, and seek a correlation between these manifestations and the *MEN1* mutational status.

## Materials and methods

### Patients

Clinical data of 106 index cases with MEN1 syndrome followed up at the Endocrine Unit of Pisa from January 1997 to May 2022 were retrospectively collected. Seventy-nine relatives carrying *MEN1* mutations were also evaluated. Data obtained up to 2015 were already reported (2). The diagnosis of MEN1 syndrome was made according to the criteria established by the latest International guidelines, namely, i) familial MEN1 (F-MEN1): the presence of at least two MEN1 major lesions in the index case, with a first-degree relative with at least one major lesion; 2) sporadic MEN1 (S-MEN1) in the absence of a family history of MEN1-related manifestations; 3) atypical MEN1 by the association of a single major lesion with one or more uncommon MEN1-related manifestations (5).

All patients underwent a total skin examination by the attending endocrinologist on the first visit to our center and repeated each follow-up visit. The clinical criterion for the diagnosis of angiofibroma was a dome-shaped, skin-colored to red papule located on the central face, usually around the nose and on the malar eminences (30). Angiofibromas were considered multiple when more than three lesions were detected. Collagenomas are benign connective tissue nevi and usually present as asymptomatic, firm, round to oval hypopigmented, or skin-colored papules preferentially located on the trunk and upper part of the arms. Lipomas were defined as non-painful, round, mobile masses, with a characteristic soft, doughty feel localized at a subcutaneous or visceral site (31). The diagnosis of cutaneous lipomas was made by clinical examination and/or ultrasonography (US), while visceral lipomas were mostly diagnosed with contrast-enhanced computed tomography (CECT), magnetic resonance imaging (MRI), or US. Lipomas that were surgically removed underwent histological examination. We also included angiolipoma, which is a variant of lipoma with co-existing vascular proliferation (32). Hibernomas are rare benign tumors originating from the brown adipose tissue, usually located in the thigh, shoulder, and back. In most cases, they are asymptomatic, although occasionally a “pressure” type of pain may be present. Hibernomas are typically mobile and pliable. Imaging plays a key role in their diagnosis (33). We detected hibernoma during clinical exams and then confirmed it by CECT. The presence of suspicious melanoma was confirmed histologically after surgical excision.

Uterine leiomyomas, if not referred to medical history, were identified by complete abdominal ultrasound and/or CECT or MRI, including uterus evaluation, routinely performed in women of any age according to the MEN1 guidelines (5). Meningiomas were incidentally identified by pituitary MRI performed during the regular follow-up and screening of pituitary adenoma or by CECT or whole-brain MRI (available in 35 out of 185 patients of the whole cohort) performed for other purposes. Breast cancer was diagnosed with US or mammography during breast cancer screening according to general population guidelines or as an incidental finding on the chest CECT scan performed for the screening of the MEN1-related tumors.

Gastric leiomyomas appeared as a well-defined solid mass with smooth contours and low homogeneous contrast enhancement on CECT (34). The diagnosis was confirmed by endoscopic ultrasound.

The study was part of the regular patient follow-up with retrospective analysis of data on the basis of the written informed consent routinely obtained from the overall patient population in the institution.

### Gene nucleotide sequence analyses

DNA was extracted from index patients’ peripheral leucocytes with Maxwell16 Instrument according to the manufacturer’s instructions (Promega Corp., Madison, WI, USA). The entire coding region and intron/exon boundaries of *MEN1* (GenBank entry NM_130799.2), *CDKN1B* (NM_004064.5), and *AIP* (NM_003977.4) genes were first investigated by sequencing germline DNA from all index patients. PCR-amplified DNA was sequenced in forward and reverse directions by direct cycle-sequencing using BigDye Sequencing Reaction kit v.1.1 (Applied Biosystems, Foster City, CA, USA) and run-on ABI 3130XL automated sequencer (Applied Biosystems). In kindreds carrying *MEN1* mutation, the mutational analysis of the region of interest was extended to first-degree relatives of the index case.

### Multiplex ligation-dependent probe amplification assay

Multiplex ligation-dependent probe amplification (MLPA) analysis has been performed on the DNA of the index cases that resulted negative by sequencing analysis to detect possible large monoallelic deletions or amplifications in *MEN1*, *AIP*, and *CDKN1B* genes. We used the SALSA MLPA probemix kit P244-C1 (MRCHolland, Amsterdam, The Netherlands). The assay was performed according to the manufacturer’s instructions, as previously reported (35). Every experiment included almost three reference DNA blood samples derived from healthy subjects that are not expected to have any copy number changes in the region of interest and a negative control sample with no DNA, as well as appropriate positive controls.

### Statistical analysis

Quantitative variables with normal data distribution were expressed as mean and standard deviation (SD). The associations between F-MEN1 and S-MEN1 or *MEN1* mutation-positive and mutation-negative patients and dichotomous variables (e.g., presence or absence of MEN1-related non-endocrine tumors) were determined using chi-square or Fisher’s exact tests, as appropriate. Multivariate exact logistic regression analyses were performed to evaluate the previously mentioned associations after adjustment for age at the last visit. A value of p < 0.05 was considered statistically significant.

## Results

### Demographic data

The whole series included 185 MEN1 patients: 106 index cases and 79 relatives. This cohort included 119 (64%) women and 66 (36%) men (female-to-male ratio of 1.8:1), with a mean age at the first manifestation of 41 years (SD ± 16, range 6–88 years). Fifty (47%) index cases were classified as F-MEN1 and 55 (53%) as S-MEN1. The remaining patient was not classified as familial or sporadic because she was adopted.

*Familial MEN1 (n = 129)*: This group included 50 index cases and 79 relatives, with a female-to-male ratio of 1.4:1 (**Table 1**). The mean age at first diagnosis was 37 years (SD ± 17, range 6–88 years).

**Table 1:** Demographic, MEN1 mutation status, and non-endocrine manifestations of MEN1 patients.

MEN1 patients	N	Age at diagnosis, years (mean ± SD)	Male, n (%)	Female, n (%)	*MEN1*-mutated, n (%)	MEN1-WT, n (%)	Patients with non-endocrine manifestations, n (%)
All index cases	106	40 ± 16	29 (27%)	77 (73%)	58^a^ (57%)	46^a^ (44%)	Cutaneous lesions^b^: 47 (44%) Lipoma, 39 (37%) Angiofibroma, 19 (18%) Basal cell carcinoma, 3 (2.8%) Melanoma, 2 (1.9%) Collagenoma, 1 (0.9%) Hibernoma, 1 (0.9%) Other associated tumors: Uterine leiomyoma, 16 (20%)^c^ Thyroid cancer, 7 (6.6%) Breast cancer, 4 (3.8%) Lung cancer, 3 (2.8%) Gastric leiomyoma, 2 (1.9%) Meningioma, 2 (1.9%) Colorectal cancer, 1 (0.9%) Neuroblastoma, 1 (0.9%)
Familial index cases	50	40 ± 13	18 (36%)	32 (64%)	47 (94%)	3 (6%)	Cutaneous lesions^b^: 31 (62%) Lipoma, 25 (50%) Angiofibroma, 15 (30%) Basal cell carcinoma, 1 (2%) Collagenoma, 1 (2%) Hibernoma, 1 (2%) Melanoma, 1 (2%) Other associated tumors: Uterine leiomyoma, 8 (25%)^c^ Thyroid cancer, 3 (6%) Gastric leiomyoma, 2 (4%) Breast cancer, 1 (2%) Meningioma, 1 (2%) Neuroblastoma, 1 (2%)
Familial relatives	79	37 ± 16	37 (47%)	42 (53%)	79 (100%)	0 (0%)	Cutaneous lesions^b^: 38 (48%) Angiofibroma, 26 (33%) Lipoma, 23 (29%) Melanoma, 2 (2.5%) Other associated tumors: Uterine leiomyoma, 10 (24%)^c^ Meningioma, 3 (3.8%) Thyroid cancer, 2 (2.5%) Breast cancer, 1 (1.3%)
Sporadic index cases	55	47 ± 14	10 (18%)	45 (82%)	10^a^ (19%)	43^a^ (81%)	Cutaneous lesions^b^: 16 (29%) Lipoma, 14 (25%) Angiofibroma, 4 (7.3%) Basal cell carcinoma, 2 (3.6%) Melanoma, 1 (1.8%) Other associated tumors: Uterine leiomyoma, 8 (18%)^b^ Thyroid cancer, 4 (7.3%) Breast cancer, 3 (5.4%) Lung cancer, 3 (5.4%) Colorectal cancer, 1 (1.8%) Meningioma, 1 (1.8%)
Adopted index case	1	54	0 (0%)	1 (100%)	1 (100%)	0 (0%)	Cutaneous lesions^b^: 0 (0%) Other associated tumors: 0 (0%)

a In two patients, genetic test is still ongoing.

b Number of patients with almost one cutaneous lesion. Both cutaneous and visceral lipomas have been included.

c Percentage was calculated in the female population.

Nine patients (mean age 23 years, range 8–62 years) had no clinical manifestations but carried *MEN1* gene mutation.

*Sporadic MEN1 (n = 55)*: In this cohort, we observed a ± higher female-to-male ratio (4:1), with a mean age at diagnosis of 47 years (SD 14, range 17–70 years) (**Table 1**).

### Endocrine manifestations

PHPT was present in 175 (95%) patients, GEP tumors in 116 (63%), pituitary adenomas in 85 (45%), and adrenal lesions in 64 (35%). Nine family members were *MEN1* gene carriers with no abnormalities in biochemical analyses or instrumental evidence of main endocrine-associated tumors. Two of them only presented non-endocrine manifestations (one angiofibroma and one melanoma).

The classical triad of MEN1-related tumors (PHPT, GEP, and pituitary) was present in 49 (28%), PHPT and GEP tumors in 67 (38%), and PHPT and pituitary tumors in 35 (20%), whereas PHPT alone or associated with minor tumors (adrenal lesions, lung and thymic carcinoids, gastrointestinal stromal tumors, and pheochromocytomas) was observed in 24 (14%) affected patients of the whole series. Thirty-one (29%) index cases presented the classical triad of MEN1-related tumors, 37 (35%), PHPT and GEP tumors, and 31 (29%) PHPT and pituitary adenomas. Seven index cases (7%) had PHPT alone or were associated with minor tumors.

*Familial MEN1 (n = 129)*: In the whole series, PHPT was present in 119 (92%) patients, GEP tumors in 90 (70%), pituitary adenoma in 45 (35%), and adrenal lesions in 43 (33%). Fifty-three (44%) patients developed PHPT and GEP tumors; 37 (31%) PHPT, pituitary, and GEP tumors; and 7 (6%) PHPT and pituitary adenomas. Twenty-two (18%) patients had PHPT with or without minor tumors, and one relative (1%) had only pituitary adenoma. Nine patients had no clinical manifestations but carried *MEN1* gene mutation.

The phenotype of the index cases consisted of the classical triad in 19 (38%), PHPT and GEP tumors in 23 (46%), and PHPT and pituitary adenomas in 3 (6%) patients. Five (10%) patients had PHPT with or without minor tumors.

*Sporadic MEN1 (n = 55)*: All patients had PHPT, alone or in association with other main MEN1-related tumors, 71% had pituitary adenoma, 45% had GEP tumors, and 38% had adrenal lesions. The classical triad was present in 12 (21%), PHPT and pituitary tumors in 28 (51%) patients, PHPT and GEP tumors in 14 (25%), and PHPT with or without minor tumors in 2 (4%).

### Non-endocrine manifestations

#### Cutaneous tumors, lipomas, and hibernomas

*Whole series (n = 185)*: Eighty-five (46%) patients had at least one cutaneous lesion, lipoma (cutaneous or visceral), and/or hibernoma. Lipoma, observed in 62 patients, was the most common non-endocrine manifestation (33.5%), which was cutaneous in 46 cases, visceral in 8, and both cutaneous and visceral in 8. Angiofibromas were present in 45 (24%) patients. Twenty-three patients had both lipomas and angiofibromas. Melanomas were present in four (2.2%) patients, two index cases, and two relatives. Basal cell carcinoma was present in three (1.6%). Collagenomas and hibernomas were each present only in one patient (0.5%). Lipomas represented the main non-endocrine manifestation in the index cases (37%), followed by angiofibromas, detected in 18% (**Table 1**).

**Figure 1:**
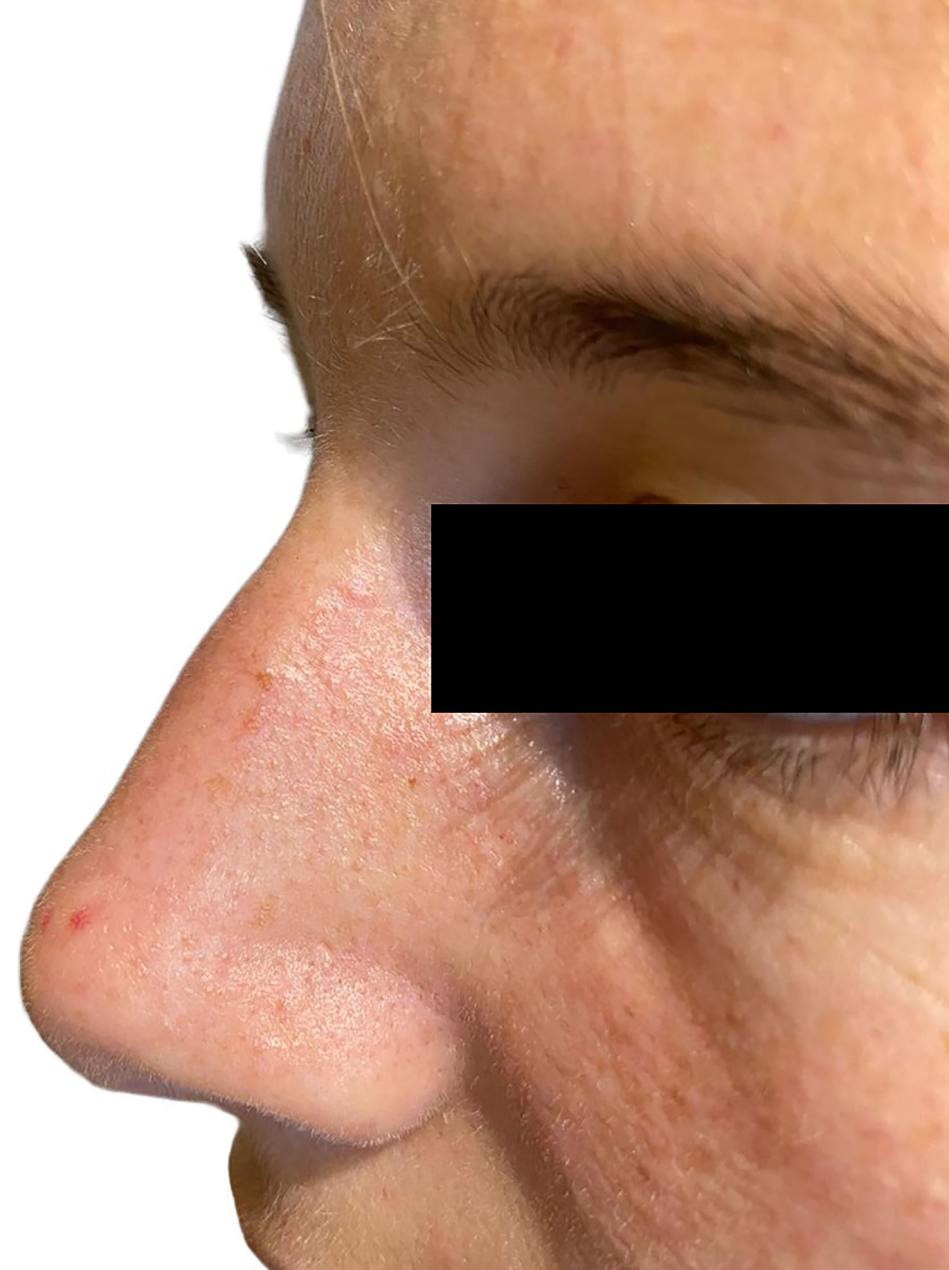
A facial angiofibroma in a 22-year-old woman with familial MEN1 is shown. It appears as a dome-shaped, skin-colored to red papule located on the nose.

**Figure 2:**
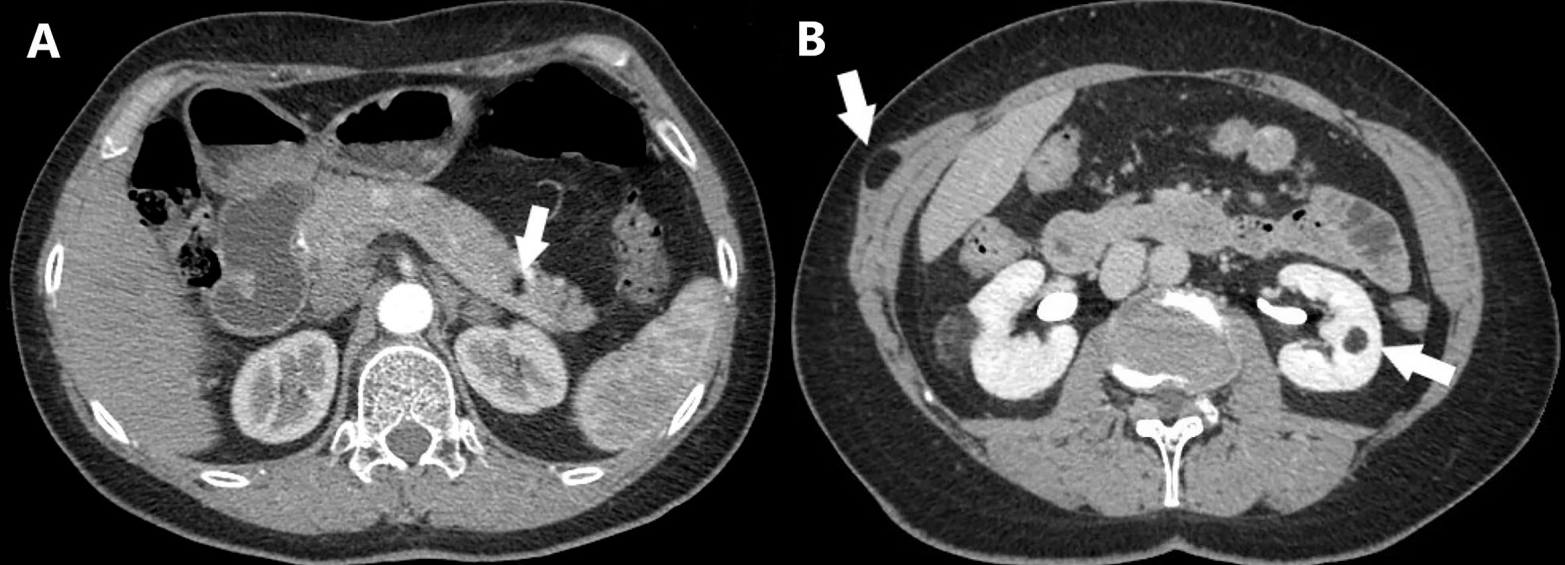
Axial CT scan of a MEN1 patient with multiple lipomatosis. **(A)** Intrapancreatic lipoma is shown (arrow). **(B)** Intramuscular lipoma and left renal angiomyolipoma are shown (arrows).

*Familial MEN1 cohort (n = 129)*: Sixty-nine (53%) patients had at least one cutaneous lesion, lipoma, and/or hibernoma. Angiofibromas were observed in 40 (31%) patients, equally distributed between index cases (30%) and relatives (33%). In 28 patients, angiofibromas were multiple and preferentially (64%) localized to the face (upper lip and nose) (**Table 2**). A representative example of angiofibroma is shown in **Figure 1**. Lipomas were observed in 48 (37%) cases, mostly detected in index cases rather than relatives (50% *vs.* 29%). Thirty-six patients had only cutaneous lipomas, five had both visceral and cutaneous, and seven had only visceral. Cutaneous lipomas had multiple localizations and were preferentially located at the thorax and the upper and lower limbs. A representative example of visceral lipoma is shown in **Figure 2**. Seventeen lipomas were surgically removed, and in one case, the histology was consistent with a malignant liposarcoma. Twenty-one (16%) patients had both angiofibromas and lipomas (visceral and cutaneous). The frequency and distribution of angiofibromas and lipomas are summarized in **Table 2**. Melanomas were present in three (2.3%) patients. Collagenoma or hibernoma was present in one (0.8%) index case.

**Table 2:** Frequency and distribution of angiofibromas and lipomas in familial and sporadic MEN1 patients.

Non-endocrine lesions	Familial MEN1 index cases (n = 50)	Familial MEN1 relatives (n = 79)	Sporadic MEN1 index cases (n = 55)
Angiofibromas, n patients *Single*, *n (%)* *Multiple*, *n (%)* Sites, n (%)	15 5 (33%) 10 (67%) Face, 13 (87%) Thorax, 2 (13%) Total, 15	26 8 (31%) 18 (69%) Face, 17 (53.1%) Thorax, 9 (28.1%) Lower limbs, 3 (9.4%) Abdomen, 2 (6.3%) Upper limbs, 1 (3.1%) Total, 32	4 1 (25%) 3 (75%) Face, 3 (75%) Thorax, 1 (25%) Total, 4
Cutaneous lipomas, n patients *Single*, *n (%)* *Multiple*, *n (%)* Site, n of lesions (%) Surgically removed, n	23 15 (65%) 8 (35%) Lower limbs, 11 (27.5%) Thorax, 11 (27.5%) Abdomen, 10 (25%) Upper limbs, 5 (12.5%) Neck, 2 (5%) Head, 1 (2.5%) Total, 40 10	18 11 (61%) 7 (39%) Upper limbs, 12 (37%) Thorax, 7 (22%) Lower limbs, 6 (19%) Head, 3 (9.4%) Abdomen, 2 (6.3%) Neck, 2 (6.3%) Total, 32 7	13 10 (77%) 3 (23%) Thorax, 8 (40%) Lower limbs, 4 (20%) Upper limbs, 4 (20%) Abdomen, 3 (15%) Neck, 1 (5%) Total, 20 2
Visceral lipomas, n patients *Single*, *n (%)* *Multiple*, *n (%)* Site, n of lesions (%) Surgically removed, n	4 3 (75%) 1 (25%) Renal, 3 (43%) Gastro-intestinal, 1 (14%) intrapericardial, 1 (14%) Hepatic, 1 (14%) Intramuscular, 1 (14%) Total, 7 2	8 6 (75%) 2 (25%) Intramuscular, 4 (40%) Gastrointestinal, 2 (20%) intrapancreatic, 2 (20%) Renal, 2 (20%) Total, 10 3	4 4 (100%) - Renal, 2 (50%) Intramuscular, 2 (50%) Total, 4 0

*Sporadic MEN1 cohort (n = 55)*: Sixteen (29%) patients had at least one cutaneous lesion and/or lipoma. Lipomas were present in 25% of the patients. None of them was surgically excised. Angiofibromas, mostly multiple, were present in 7% of the patients. Half of the patients bearing angiofibromas also had cutaneous lipomas. The frequency and distribution of angiofibromas and lipomas are summarized in **Table 2**. Melanoma was present in one (1.8%) patient (**Table 1**).

#### Smooth muscle tumors

*Whole series (n = 185)*: Uterine leiomyomas were present in 26 (22%) out of 119 women and gastric leiomyomas in two (1.1%) patients of the whole series.

*Familial MEN1 (n = 129)*: Uterine leiomyomas were observed in 18 (24%) out of 119 women, and such percentage was similar in both index cases and relatives (**Table 1**). Gastric leiomyomas were present in two (1.5%) index cases.

*Sporadic MEN1 (n = 55)*: Uterine leiomyomas were present in nine (20%) out of 45 women.

#### Central nervous system tumors

*Whole series (n = 185)*: Meningiomas were detected in five (2.7%) patients. Neuroblastoma was present in only one patient (0.5%).

*Familial MEN1 (n = 129)*: Meningiomas were present in four (3.1%) patients. Neuroblastoma was present in only one index case (0.8%).

*Sporadic MEN1 (n = 55)*: Meningioma was present in one (1.8%) patient (**Table 1**).

#### Breast cancer

In the whole series, breast cancer was present in five (2.7%) patients: one familial index case, one relative, and three sporadic cases. The median age of diagnosis was 49 years, and only one case was triple-negative invasive ductal carcinoma with lymph node metastasis with recurrence occurring 15 years later. All the remaining were unilateral and unifocal ductal carcinoma *in situ*. All patients had PHPT; however, none of them had prolactinoma or insulinoma. Seventy-five percent of them expressed the estrogen receptor. In one case, these data were not available.

### Other malignant and benign non-endocrine tumors

In the whole series, thyroid cancers (one Hurthle cell, seven papillary, and one medullary) were found in nine (4.9%), lung cancers in three (1.6%), and colorectal cancer in one (0.5%). Lung and colorectal cancers were exclusively found in sporadic cases (**Table 1**).

### Genetic analyses

**Figure 3:**
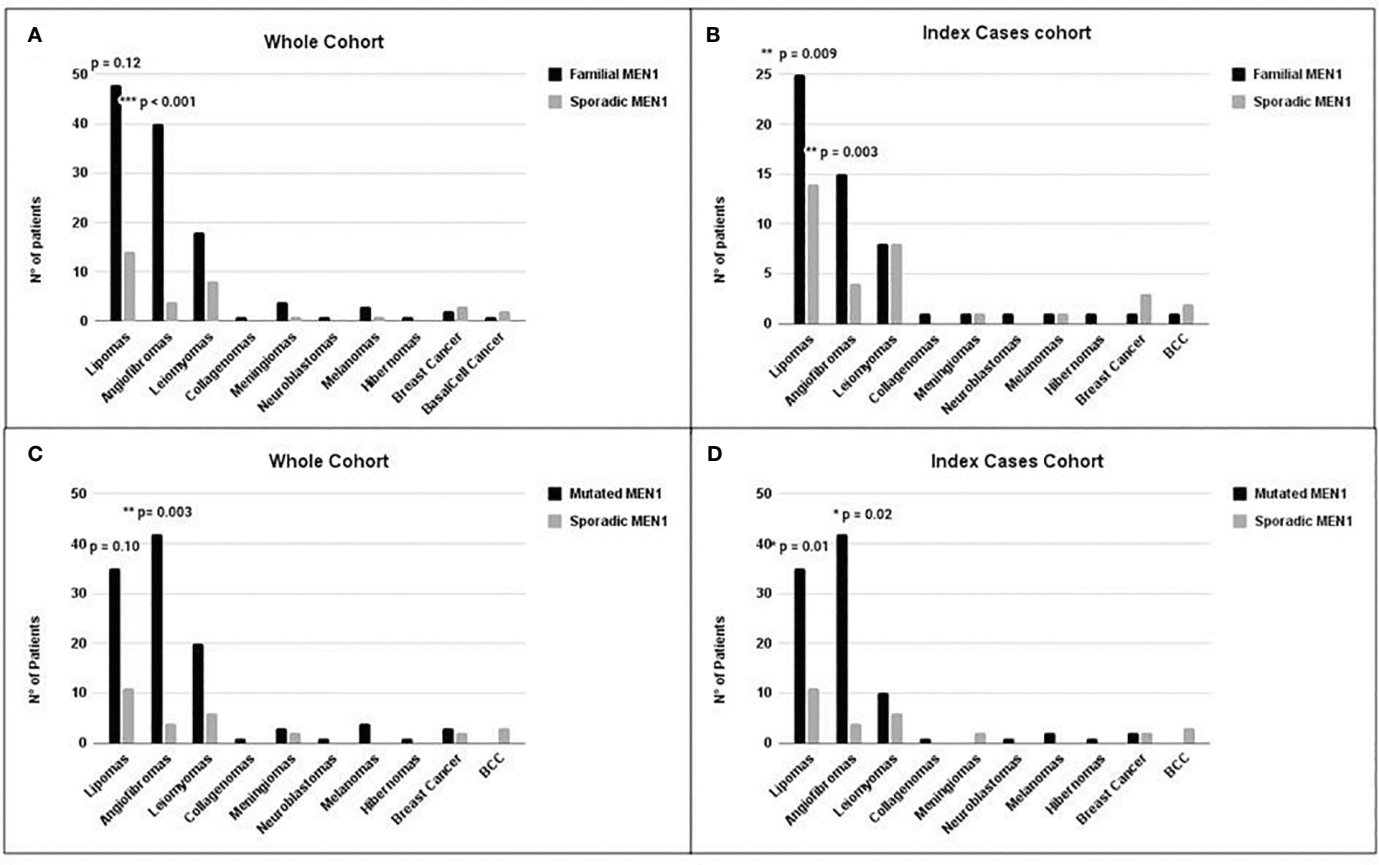
Distribution of the main non-endocrine manifestations in *MEN1* cohorts. Comparison between non-endocrine manifestations in the F-MEN1 vs. S-MEN1 in the whole cohort **(A)** and index cases **(B)** and in *MEN1* mutation-positive *vs. MEN1* mutation-negative in the whole cohort **(C)** and index cases **(D)**. Statistical significance was determined by Fisher’s or chi-square test. *p < 0.05, **p < 0.01 and ***p < 0.001.

**Figure 4:**
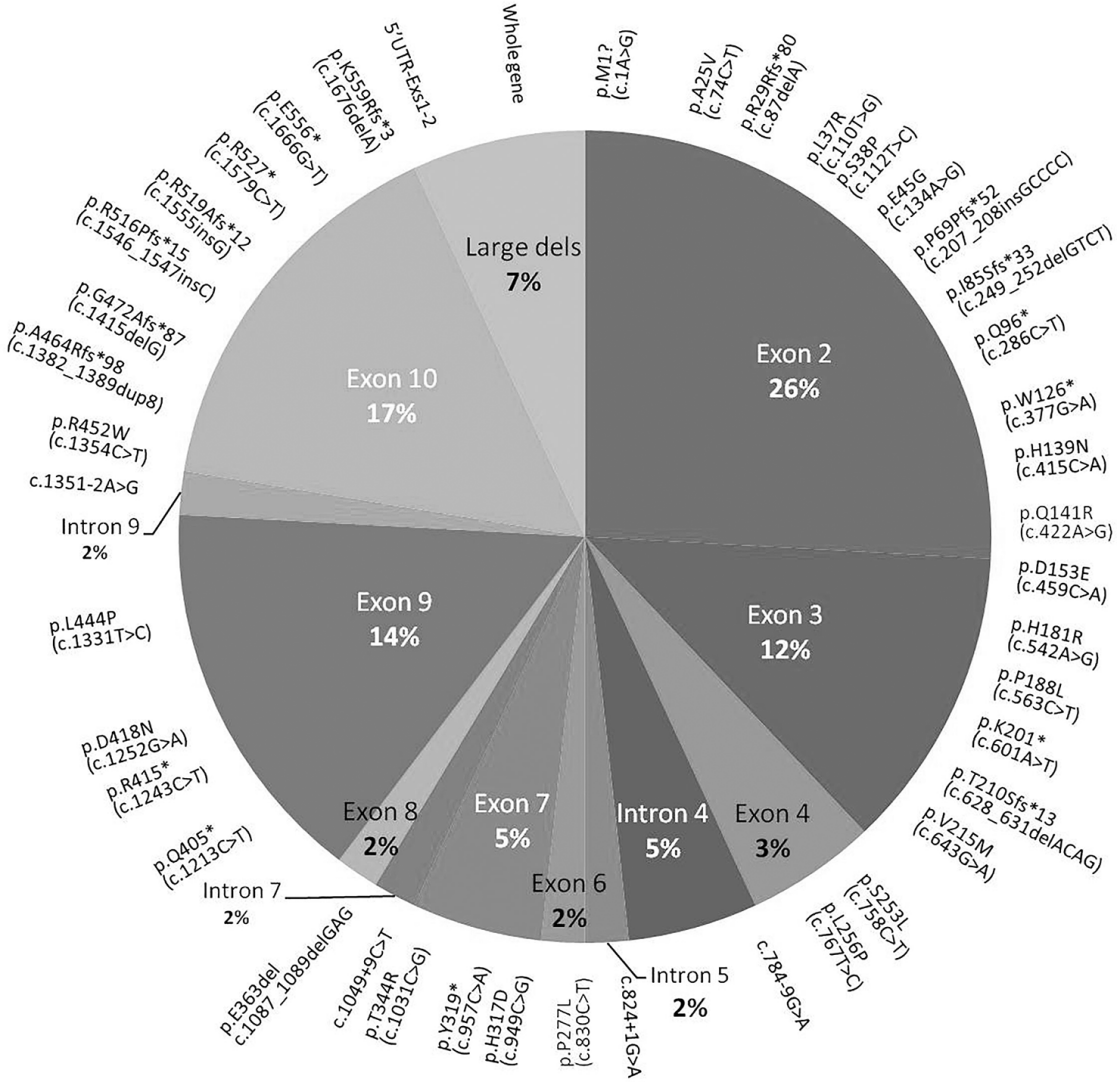
Pie chart showing the distribution of *MEN1* mutations through the exonic and intronic portions of *MEN1* gene. Mutations detected in more than one patient or one or more sporadic patients are reported in parentheses. F, familial; S, sporadic.

Some of the *MEN1* mutations identified were previously described (2). Fifty-eight index cases (56%) carried germline *MEN1* variants. DNA of two patients of the S-MEN1 cohort was not available because the patients refused the genetic test. Fifty-four variants were identified by direct sequencing and mainly localized in exons 2 (26%), 10 (17%), and 9 (14%). No mutations were detected in exon 5, and only one was detected in exons 6 and 8 (**Figure 3**). Four germline *MEN1* large deletions were identified in two F-MEN1 and two S-MEN1 cases (**Figure 4**) (2). Eight variants recurred in two or more index cases (14%). Eighty-one percent of all detected variants were identified in familial cases, being identified in 94% of F-MEN1 and 19% of S-MEN1 (p < 0.00001). Sixty percent of the variants were frameshift, non-sense, or splice site junction mutations, leading to a truncated menin protein (**Figure 4**). All but three mutations had been already described and had a proven or predicted pathogenicity. The variant p.A25V is considered a variant of unknown significance by ClinVar, although *in silico* tools (Fathmm, MutationTaster, PolyPhen-2, and Align-GVGD) all predicted a likely pathogenic role. A putative pathogenic role was also predicted for the two novel missense mutations (p.L37R and p.H317D), whose codons were already described to be affected by different substitutions (36, 37). The classifications for these three variants were further analyzed using the standards and guidelines published by the American College of Medical Genetics and Genomics (ACMG) and the Association for Molecular Pathology (AMP) that propose a clue for the interpretation of missense variants dividing them into five categories based on evidence obtained through population data, computational, functional, and segregation data and expert opinion, and workgroup (38). Varsome, according to the ACMG/AMP guidelines, reported the variant p.A25V with moderate evidence of pathogenicity (PM1 and PM2), p.L37R as likely pathogenic (PP3, PM1, and PM2), and p.H317D as pathogenic (PM5, PP3, PM1, and PM2).

One germline missense variant in exon 1 of the *AIP* gene (p.R9Q) was already reported in one S-MEN1 proband (2). Due to the conflicting interpretation of pathogenicity based on structural, functional, and *in silico* studies, this variant is reported as having uncertain clinical significance (PP5, BP1, and BP4, according to the ACMG/AMP guidelines).

### Comparison between the phenotype of non-endocrine manifestations of F-MEN1 and S-MEN1

The female-to-male ratio differs between the two cohorts (p = 0.001) with a higher female-to-male ratio in the sporadic than in the familial cohort (4.5:1 *vs.* 1.4:1). This different ratio was confirmed also in the index cases cohort (4.5:1 *vs.* 1.6:1).

We compared the prevalence of non-endocrine manifestations in F-MEN1 and S-MEN1 in the whole and index case cohorts. The prevalence of angiofibromas was significantly higher in the whole F-MEN1 cohort compared to S-MEN1 (OR = 5.9, p < 0.001) even after adjustment for age (adj. p = 0.002, data not shown). Such difference was statistically significant even when considering only index cases (OR = 5.5, p = 0.003) (**Figures 3A, B**). The prevalence of lipomas did not differ between the two whole cohorts (p = 0.12) (**Figure 3A**), but the difference between F-MEN1 and S-MEN1 became significant after adjustment for age (OR = 2.4, adj. p = 0.03, data not shown) or if we only considered the index cases cohort (OR = 2.9, p = 0.009) (**Figure 3B**). The prevalence of other minor MEN1-related non-endocrine manifestations did not differ between the two cohorts (**Figure 3**).

### Comparison between non-endocrine manifestations and *MEN1* mutational status

In this analysis, we compared the prevalence of non-endocrine manifestations in *MEN1* mutation-positive and *MEN1* mutation-negative index cases and the whole series independently of whether they were classified as F-MEN1 or S-MEN1.

In the whole cohort of familial cases, we observed a higher prevalence of angiofibromas in *MEN1* mutation-positive compared to *MEN1* mutation-negative (OR = 4.5, p = 0.003) (**Figure 3C**), also after adjustment for age. The prevalence of lipomas did not differ between the two whole cohorts (p = 0.10): however, after adjustment for age, such prevalence in *MEN1* mutation-positive was significantly higher compared to *MEN1* mutation-negative (OR = 2.7, p = 0.02, data not shown). No difference was found in the prevalence of other manifestations between the two cohorts.

In the index cases cohort, we observed a significantly higher prevalence of angiofibromas (OR = 3.7, p = 0.02) and lipomas (OR = 3.0, p = 0.01) in the *MEN1* mutation-positive compared to *MEN1* mutation-negative index cases (**Figure 3D**). No difference was found in the prevalence of other minor MEN1-related non-endocrine manifestations between the two cohorts.

We further conducted two additional analyses: we compared the occurrence of non-endocrine lesions in *MEN1* mutation-positive index cases of F-MEN1 (n = 47) *vs.* S-MEN1 (n = 10) and in *MEN1* mutation-positive (n = 10) *vs.* mutation-negative (n = 43) index cases of S-MEN1. No significant differences were found in the prevalence of any non-endocrine lesions in both comparisons.

## Discussion

The association between a variety of cutaneous lesions (i.e., angiofibromas, collagenomas, and lipomas) and MEN1 syndrome was first reported in 1997 by Darling et al. (20) and has been confirmed in subsequent studies (39, 40). Biopsies of facial angiofibromas, lipomas, and collagenomas from patients harboring germline *MEN1* variants exhibited an allelic deletion of chromosome 11 including the *MEN1* locus (24). This alteration was specific to these lesions and absent in other skin lesions found in the same patients but not typically associated with the syndrome (24). Rusconi et al. showed that in a patient carrying germline heterozygous *MEN1* mutation, the somatic inactivation of the wild-type allele arose in different MEN1-related tumors (pituitary adenoma and lipoma) of the same patient by distinct mechanisms, i.e., loss of heterozygosity and balanced translocation (41).

Cutaneous manifestations are often underestimated or considered ancillary findings not related to the classical clinical spectrum of the MEN1 syndrome, as they are typically benign and do not usually require specific treatments. Only six previous studies evaluated the frequency of cutaneous lesions in a series of patients with MEN1 (20–22, 25, 42, 43). In a prospective study including 110 consecutive patients with gastrinomas, either sporadic or in the context of MEN1 syndrome, the combined presence of multiple angiofibromas and any collagenomas was considered the best cutaneous diagnostic criterion for MEN1 with a specificity of 95% and a sensitivity of 75% (22).

In our study, 46% of patients of the whole series, including relatives carrying *MEN1* mutation, had at least one cutaneous lesion, lipoma, and/or hibernoma. These lesions were significantly more frequent in familial *vs.* sporadic cohorts. An overall prevalence of cutaneous lesions in MEN1 patients reported in the literature ranges from 22% to 55% (21, 42, 43). Our results are consistent with those reported by Vidal and colleagues (13) in nine *MEN1* mutation-positive patients. They compared the prevalence of cutaneous lesions (angiofibromas, collagenomas, melanosis guttaca, lipomas, melanomas, and “café-au-lait macules”) with 20 non-carrier relatives and found a significantly higher prevalence of any cutaneous lesions in the former compared to the latter group (55.5% *vs.* 25%; p = 0.029) (21).

Angiofibromas, mostly multiple (69%) and localized to the face (upper lip and nose), were present in about one-quarter (24%) of the whole series and 18% of the index cases. The prevalence of angiofibromas in MEN1 among different studies ranges from 0% to 88% (12–14, 17, 32, 33). This wide difference might be due to the expertise of the skin examiner. Of interest, the three studies in which experienced dermatologists performed a thorough skin evaluation reported a prevalence of angiofibromas of 88%, 65%, and 22% (20–22).

The prevalence of angiofibromas in our *MEN1* mutation-positive patients was significantly higher compared to that of *MEN1* mutation-negative. This finding supports the hypothesis that mutations in *MEN1* gene could lead to the abnormal proliferation of some cutaneous cells and the development of skin lesions reported in patients with the syndrome (24). The significantly higher prevalence of angiofibromas in our familial compared to sporadic MEN1 cohort, observed in both the whole and index cases, was expected due to the association between *MEN1* mutations and familial MEN1 cases (94% F-MEN1 mutation*-*positive *vs.* 19% S-MEN1 mutation*-*positive, p < 0.00001).

The presence of lipomas in patients with MEN1 has been reported since the first description of the syndrome (11, 44). An *in vitro* study that matched normal and menin-deficient adipocytes from wild-type and menin-null mouse embryonic stem cells showed that menin deficiency led to fat-cell hypertrophy, supporting the causal relation between *MEN1* gene alterations and the onset of lipomas (45). In our study, the prevalence of lipomas (33.5%) is in agreement with that reported in the literature (20–22, 43, 46–50). We found a significantly higher prevalence of lipomas in *MEN1* mutation-positive compared to *MEN1* mutation-negative (p = 0.01) and in familial compared to sporadic (p = 0.009) cohorts if only index cases were considered. Although a clear correlation between the prevalence of lipomas and patient age or disease duration was not demonstrated through literature (22), our data suggest that the young age (46 ± 19 *vs.* 57 ± 14) and/or a short follow-up (11 *vs.* 21 years) of relatives compared to index cases might contribute to the underestimation of the prevalence of these lesions.

The lack of a significant difference in the prevalence of any non-endocrine lesions between *MEN1* mutation-positive index cases of the F-MEN1 (n = 47) and the S-MEN1 (n = 10) cohort seems to confirm previous findings, suggesting a putative role of *MEN1* mutations in the classic phenotype of MEN1 syndrome, including the occurrence of related non-endocrine manifestations.

The observed difference in the prevalence of two main non-endocrine manifestations between *MEN1* mutation-positive and mutation-negative patients, as well as familial and sporadic index cases, raises the central issue about the existence of MEN1 phenocopies. Among the group of S-MEN1 patients who tested negative for *MEN1* mutations (80%), 82% had two main MEN1-related lesions (70% of them had PHPT and pituitary adenoma, and 30% had PHPT and GEP), whereas the remaining 18% had the classical triad. These patients may have the so-called MEN1 phenocopies, which are characterized by a later onset of the first manifestation, a lower likelihood of developing a third MEN1-related lesion, and a life expectancy comparable with that of the general population (2, 8, 13–15). Mutations of other genes (*CDKN1B* and other CDKI genes, *CASR*, *AIP*, and *CDC73*) might be responsible for a MEN1-like phenotype (2, 16–19). For this reason, all patients also underwent Sanger sequencing for *CDKN1B* and *AIP* genes. Only one S-MEN1 patient carried an already reported germline missense mutation (p.R9Q) in *AIP* gene (2, 51, 52). This patient presented a triad consisting of multiglandular PHPT, non-functional GEP, and a pituitary adenoma co-secreting PRL and GH, a type of pituitary tumor common in *AIP* mutation-positive patients with familial isolated pituitary adenoma (53). Due to the conflicting interpretation of pathogenicity based on structural, functional, and *in silico* studies, this variant is reported of uncertain clinical significance.

Nevertheless, a possible reason for false-negative genetic tests might be due to technical problems: Sanger sequencing, theoretically a very sensitive method for heterozygous germline mutations, might have missed mutations, especially those present in the sequencing traces as well as genetic mosaicisms (54). Moreover, although the prevalence of alterations in 5′ and 3′ untranslated regions as well as in introns seems to be very low (55, 56), we cannot exclude such events. Of note, three F-MEN1 patients had no mutations of *MEN1*, *CDKN1B*, and *AIP* genes. One of them had the classical triad, namely, PHPT, pituitary adenoma secreting GH, pancreatic non-secreting tumor, adrenal adenoma and angiofibroma, lipoma, meningioma, and uterine leiomyomas. The remaining two index cases had PHPT and prolactinoma, and one of them also had an adrenal adenoma. We can hypothesize that the genetic standard analyses may have missed the rare anomalies in non-codifying DNA regions (highly likely for the first patient) or that they represent MEN1 phenocopies.

To address the question of whether the higher female-to-male ratio in the sporadic than in the familial cohort might account for the difference in the prevalence of skin lesions, we checked for a potential gender difference in the prevalence of angiofibroma and lipoma in the general population. No gender difference in the prevalence of angiofibroma was reported in the literature (57). To our knowledge, no data on gender differences were available for lipoma. We evaluated the prevalence of lipomas in men and women in our cohort of 185 patients. We found no statistical difference in the prevalence of lipoma between gender (women, 26%; men, 35%; p = 0.17). Thus, we may conclude that the different female-to-male ratios observed between familial and sporadic cohorts would not account for the statistical difference in the prevalence of such lesions.

Of interest, in one familial *MEN1* mutation-positive index case, the histologic examination of an apparently resected benign lipoma was consistent with the diagnosis of liposarcoma. To our knowledge, only two cases of liposarcoma were reported in patients with MEN1 (58, 59).

We found one case of hibernoma under the left scapula in a relative of familial *MEN1* mutation-positive. Only seven cases of hibernoma in MEN1 patients have been reported in the literature (60–65), and a relationship between this manifestation and the syndrome seems to be supported by genetic analysis (60, 64). The discovery of deletions of *MEN1* gene in resected hibernomas of MEN1 and non-MEN1 patients supported the role of this gene in the development of these lesions (66). Later studies underlined that deletions involved a large region on 11q13 also including the gene encoding aryl hydrocarbon receptor-interacting protein (AIP), and the concomitant loss of *MEN1* and *AIP* were supposed to be involved in the pathogenesis of hibernoma (67, 68).

We also reported only one case of collagenoma in a familial MEN1 index case. The prevalence of collagenoma in MEN1 reported in the literature ranges from 0% to 72% (20–22). Pack and colleagues found 11q13 loss of heterozygosity in collagenomas resected from MEN1 patients, which was not present in other skin lesions unrelated to the syndrome, supporting the association of this manifestation with MEN1 (24).

Three *MEN1* mutation-positive and one *MEN1* mutation-negative patients had melanomas. Melanomas were reported in other MEN1 cohorts, but a clear relationship between these lesions and *MEN1* gene alteration has not been proved (39, 40). Although some studies reported a tumor suppressor role for *MEN1* in sporadic melanomas, the relatively high prevalence of this malignancy in the general population and the lack of evidence of a somatic hit in resected melanoma of MEN1 patients suggest that the occurrence of melanomas in MEN1 syndrome may be incidental (69, 70).

Neoplasms of the central nervous system, i.e., meningiomas, ependymomas, and schwannomas, have been reported as clinical manifestations of MEN1 syndrome (5). Asgharian et al. suggested that the increased occurrence of meningiomas in MEN1, observed in previous studies, was not accidental. They found that meningiomas were 11 times more frequent in patients with MEN1 and Zollinger–Ellison (ZES) syndrome than with ZES alone and demonstrated that allelic loss at *MEN1* but not at *NF2* gene locus and frequent alterations in sporadic meningiomas play a role in the pathogenesis of MEN1-associated meningioma (71). Herein, meningioma was present in 5/185 (2.7%) cases: three familial *MEN1* mutation-positive, one familial, and one sporadic *MEN1* mutation-negative patients. The co-occurrence of meningioma and pituitary adenoma, especially GH-omas, has been reported (72, 73). A genetic predisposition, i.e., a germline *MEN1* mutation, seems to explain the high rate of the simultaneous development of these two benign tumors of the central nervous system (74). In our study, only one patient with meningioma also had a GH-oma.

Leiomyomas are benign mesenchymal smooth muscle tumors that arise throughout the body but most commonly affect the uterus, representing the most common neoplasms of reproductive-aged women (75). Although an association between leiomyoma and MEN1 had been previously suggested by several case reports, Vortmeyer and colleagues first demonstrated the inactivation of *MEN1* gene in the esophageal leiomyoma tissue of a MEN1 patient, suggesting that this neoplasm could share a common molecular cause with the main MEN1-associated tumors (76). Loss of heterozygosity at *MEN1* locus was also found in esophageal and uterine leiomyomas in four of five F-MEN1 patients, whereas such loss seemed not to play a role in sporadic uterine leiomyomas (77). In our study, 22% of women developed uterine leiomyomas. A systematic review of the general population reported an incidence range between 5.4% and 77% in women of reproductive age (78). Due to this wide range, a comprehensive comparison between our data and those of the literature is difficult. Nevertheless, we calculated the cumulative incidence of leiomyoma in our MEN1 patients across age groups. An increase in the cumulative incidence of leiomyoma was observed according to the age groups (2.9% in women <30 years, 5.7% between 30 and 39 years, 13.9% between 40 and 49 years, and 27% >50 years). A similar trend of increase but with higher cumulative-incidence events was reported by Baird et al. in Caucasian women (79). Therefore, we may speculate that the association between MEN1 syndrome and uterine leiomyoma remains still to be established.

Only one study has carried out a systematic evaluation of such lesions and reported a prevalence of 12.6% in the MEN1 female population (43).

Five women in our whole series (4.2% of the female population) developed breast cancer. In recent years, there has been a growing interest in the relationship between breast cancer and MEN1 syndrome (27, 28, 40, 80). To date, breast cancer (approximately ninety cases) has been reported only in women with MEN1 (40). Among four unrelated cohorts from Holland, the United States, Tasmania, and France, which respectively included 190, 68, 71, and 536 women affected by MEN1 with a follow-up from 5 to 27 years, breast cancer has 2.3- to 2.8-fold penetrance than the control population, obtained from the respective national cancer registries (81). A reduced menin staining in breast cancer samples and loss of heterozygosity at the *MEN1* locus in one-third of *MEN1*-mutated patients with breast cancer was reported, suggesting a possible role of the gene in breast carcinogenesis (81). Given the increased risk, a Dutch study suggested starting breast cancer screening from the age of 40 in MEN1 women (27). Since there is currently no dedicated screening program for women with MEN1, it is important to inform them about the potential increased risk and provide appropriate counseling.

Our study has several strengths: i) a large cohort of consecutive patients with MEN1 syndrome having full clinical, biochemical, instrumental, and genetic characterization followed up at a single Italian endocrine outpatient clinic; ii) inclusion of a large cohort of sporadic MEN1 cases; iii) comparison between the clinical characteristics of patients with sporadic and familial MEN1 syndrome; iii) inclusion of a relatively high number of *MEN1* mutation-negative patients that allowed a statistical comparison of the prevalence of non-endocrine manifestations between *MEN1* mutation-positive and *MEN1* mutation-negative to strengthen the putative pathogenic role of *MEN1* mutations in the development of some cutaneous lesions. However, our study does have some limitations: i) difficulty in assessing the true incidence of *de novo* mutations in the sporadic MEN1 cohort due to the unavailability of genetic data of index case’s parents; ii) the lack of systematic evaluation of the skin manifestations by a dermatologist that might have underestimated the presence of some cutaneous lesions, i.e., collagenomas; iii) the lack of the study of the whole brain with MRI or CECT might have underestimated the presence of meningiomas or ependymomas.

In conclusion, the results of our study contribute to increasing knowledge regarding non-endocrine manifestations of MEN1 syndrome. We found a significantly higher prevalence of angiofibromas and lipomas in F-MEN1 compared with S-MEN1. Both these manifestations were significantly more frequent in *MEN1* mutation-positive compared to *MEN1* mutation-negative patients.

In patients presenting with one major endocrine manifestation of MEN1, the presence of cutaneous lesions, i.e., angiofibromas and lipomas, might suggest the diagnosis of MEN1 and a possible indication of genetic screening. In these patients, it is advisable to perform an accurate physical examination, which includes a total skin examination to identify the presence of these manifestations.

Further studies are necessary to describe all non-endocrine manifestations of the syndrome and tailor a personalized approach during the follow-up and screening of some malignancies such as breast cancer.

## Data availability statement

The original contributions presented in the study are publicly available. Genomic data can be found in LOVD v. 3.0 (https://www.lovd.nl/), gnomAD v. 2.1 (https://gnomad.broadinstitute.org/), dbSNP (https://www.ncbi.nlm.nih.gov/snp/), dbVAR (https://www.ncbi.nlm.nih.gov/dbvar/). The raw data supporting the conclusions of this article will be available from the corresponding author on reasonable request.

## Ethics statement

The studies involving human participants were reviewed and approved by Comitato Etico Regionale per la sperimentazione clinica - Sezione autonoma Area Vasta Nord Ovest (CEAVNO). Written informed consent to participate in this study was provided by the participants’ legal guardian/next of kin.

## Author contributions

LP, CM, and FC contributed to the study conception and design and wrote the first draft of the manuscript. AM, MC, EP, and SB performed the genetic analyses. LP, EP, ED, SB, SD, CS, and FB collected all the data. PP performed the statistical analyses. LP, EP, CM, and FC revised the manuscript. All authors read and approved the final manuscript. FC is a co-coordinator of the Mineral and Bone Club of the Italian Society of Endocrinology (part of research projects). All authors contributed to the article and approved the submitted version.
